# The evaluation of nasolacrimal duct injury in Le Fort I osteotomy patients

**DOI:** 10.4317/medoral.26167

**Published:** 2023-08-25

**Authors:** Yusuf Nuri Kaba, Ahmet Emin Demirbas, Cihan Topan, Canay Yılmaz-Asan, Nihal Ersu

**Affiliations:** 1 Lecturer, DDS, Department of Oral and Maxillofacial Surgery, Erciyes University Faculty of Dentistry, Kayseri, Turkey; 2 Associated Professor, DDS, PhD, Department of Oral and Maxillofacial Surgery, Erciyes University Faculty of Dentistry, Kayseri, Turkey; 3 Assistant Professor, DDS, PhD, Department of Oral and Maxillofacial Surgery, Erciyes University Faculty of Dentistry, Kayseri, Turkey; 4 Resident, DDS, Department of Oral Radiology, Erciyes University Faculty of Dentistry, Kayseri, Turkey

## Abstract

**Background:**

Although Le Fort I surgeries are safe and successful procedures; nasolacrimal duct injuries may be observed due to these surgeries. The study aimed to investigate the prevalence of nasolacrimal duct injury in Le Fort I osteotomy patients.

**Material and Methods:**

The authors conducted a retrospective cohort study consisting of patients who underwent Le Fort I osteotomies between 2017 and 2021 in the Erciyes University Faculty of Dentistry. The primary predictor variables were the distance of the nasolacrimal canal to the outer cortex of the maxilla and the nasal floor, as well as the superior-inferior level of the superiorly positioned screw inserted in the maxilla aperture region relative to the nasolacrimal canal. The outcome variable was the presence of a nasolacrimal duct injury. Mann Whitney U test was used for quantitative variables between the two groups. A Pearson chi-squared analysis was used to compare categorical data. A *p-value* <0.05 was considered statistically significant.

**Results:**

A total of 290 nasolacrimal canals were evaluated in 145 patients, 87 females, and 58 males. The mean age was 23.47± 6.67. There was a statistically significant relationship between screw level and nasolacrimal canal perforation (*p*<0,001). The distance between the most anterior border of the nasolacrimal canal and the outer cortical of the maxilla was significantly less in the perforation group (*p*<0,001). The fixation screw was significantly closer to the nasolacrimal canal in the perforation group (*p*<0,001).

**Conclusions:**

In Le Fort I surgery, nasolacrimal duct injury may occur during screw fixation to the aperture region. Superiorly positioned fixation screws in the aperture region may damage the nasolacrimal canal. In patients where the nasolacrimal canal is close to the outer cortex, care should be taken when applying the fixation screws to the aperture region to avoid damaging the canal.

** Key words:**Le Fort 1 osteotomy, nasolacrimal canal injury, fixation.

## Introduction

Le Fort I osteotomy is a safe and successful surgical procedure that is widely used to correct skeletal maxillary orthognathic deformity. Although Le Fort I osteotomy is often a predicTable operation, the complications associated with this procedure are well documented in the literature (1-5). These complications can be summarized as unwanted fractures, hemorrhagic complications, infection, necrosis, nasal septal deviation, neurosensory disorders, malunion, and nonunion. The incidence of complications during Le Fort I osteotomies varies between 4% and 9% (1,3,6). Many of these complications are not life-threatening, but rarely, deep vein thrombosis, blindness, fatal hemorrhagic problems, and aseptic necrosis may occur.

Lacrimal system injury is a rare complication after facial trauma, craniomaxillofacial surgery, rhinoplasty, nasal osteotomy, and Caldwell-Luc procedures accompanied by a nasal antrostomy. The anterior wall of the lacrimal sac and the distal aperture of the nasolacrimal duct is particularly vulnerable to inadvertent injury during craniofacial surgery (7). Nasolacrimal duct injury is another complication that occurs rarely during the Le Fort osteotomy. Nasolacrimal duct obstruction (NLDO) has been rarely reported in the literature, and it may cause permanent deterioration of the nasolacrimal system (1,8). Rarely, nasolacrimal system disruption following surgery results in a permanent obstruction, causing persistent epiphora or recurrent dacryocystitis. The Le Fort I surgery procedures that involve the superior repositioning of the maxilla, inferior turbinectomy, the insertion of osteosynthesis screws, or high Le Fort I osteotomy techniques are more likely to result in nasolacrimal duct injury (7). There are limited studies on the relationship between nasolacrimal canal injuries and Le Fort I osteotomies. Several cadaver studies are being conducted to investigate the anatomical characteristics of the nasolacrimal canal and the possibility of injury following a high Le Fort I osteotomy. The anatomy of the nasolacrimal duct in the general population has also been the subject of radiologic research. However, there is no study in the literature investigating the relationship between the nasolacrimal canal injury and the fixation screws placed during Le Fort I surgery. The authors hypothesized that screw drills or the screw itself could cause injury to the nasolacrimal duct. This is most likely to occur during the insertion of osteosynthesis screws and the superior positioning of the maxilla. Especially in such cases where the distal part of the nasolacrimal duct is too low and close to the outer cortical wall, nasolacrimal duct damage may occur during the placement of the screws in the aperture.

The retrospective study aimed to investigate the prevalence of nasolacrimal duct injury due to fixation drills and screws during the Le Fort I osteotomy. The study also aimed to investigate the risk factors arising from the anatomical nature of the nasolacrimal canal.

## Material and Methods

- Study design

The authors designed a retrospective cohort study for 145 patients who underwent Le Fort I osteotomies at Erciyes University Faculty of Dentistry, Department of Oral and Maxillofacial Surgery, between 2017 and 2021. A written exemption was granted by the Erciyes University IRB due to the retrospective nature of the study (Decision No. 2022/406). Informed written consent was obtained for all volunteers. The study comprised patients who were older than 18 and had complete preoperative and postoperative cone beam computed tomography (CBCT) data. Patients with a history of craniofacial injuries, tumors, or other pathology were excluded from the study. Patients without preoperative records or CBCT images were also excluded from the study.

- Study variables

The primary predictor variables were the distance of the nasolacrimal canal to the outer cortex of the maxilla and the nasal floor, as well as the superior-inferior level of the superiorly positioned screw inserted in the maxilla aperture region relative to the nasolacrimal canal. Primary predictive variables were evaluated with data from CBCT images of the patients. The other predictor variables were the demographic properties of the patients. The primary outcome variable was the presence of a nasolacrimal duct injury. The primary outcome variable was determined by measuring the distance between the top screw and the nasolacrimal duct.

- Radiologic scans

Preoperative CBCT images were taken at 110 kV, 5.46 mA, [16x18] FOV, 4.8 sec. irradiation time, 42.99 mAs, and 0.250 mm slice thickness (Newtom 5G, QR, Verona, Italy). The images and anatomical measurements were processed with 3D imaging software (NNT Viewer 9.1, Newtom, Verona, Italy). Preoperative and postoperative CBCT scans of all patients were obtained within 1 week.

- Preoperative radiological anatomical measurements

All the measurements were performed in a darkroom using a Dell Precision T5400 workstation (Dell, TX, USA) with a 32-inch Dell LCD screen with a resolution of 1280 x 1024 pixels. All the observers were blind to the informational subjects. One of the observers had eight years of experience, while the other had three. In the NNT program, a multiplanar reconstruction (MPR) system may generate real-time two-dimensional (2D) images of the sagittal, coronal, axial, and oblique planes.

**Figure 1:**
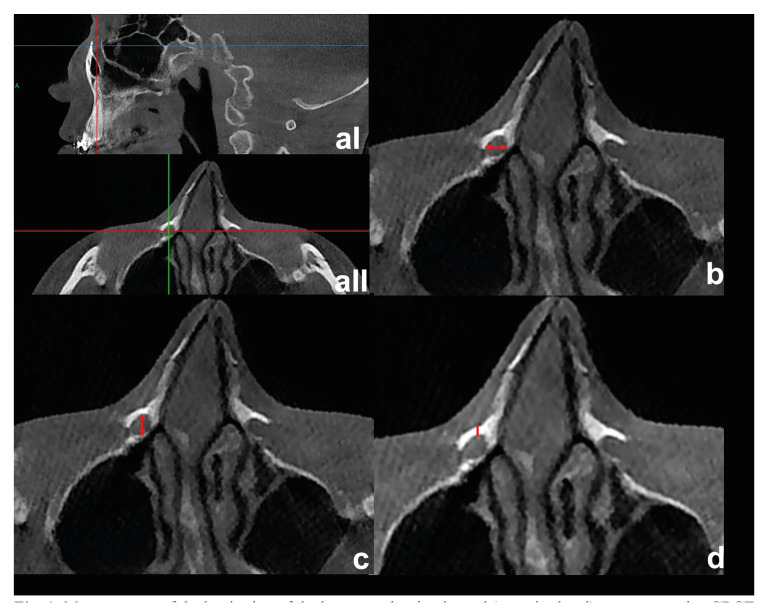
Measurement of the beginning of the bony nasolacrimal canal (superior level) on preoperative CBCT images (aI): CBCT section with standardization before measurements, aII: This section was considered the beginning of the canal, b: The mediolateral diameters of the bony nasolacrimal canal in the section where the canal begins in the axial plane, c: The anteroposterior diameter of the bony nasolacrimal canal, d: The distances between the anterior border of the nasolacrimal canal and the outer cortical surface of the maxilla.

To ensure standardization in all images, initially, the Frankfort horizontal (FH) plane was aligned parallel to the floor in the sagittal plane, and the line passing through the bilateral frontozygomatic suture was also aligned parallel to the floor in the coronal plane (Fig. 1).

The first section is where the cortical bone border integrity of the canal is provided in the axial plane. This section was considered the beginning of the canal (Fig. 1). The integrity of the cortex was checked in the sagittal, axial, and coronal planes. The last section in which the cortical bone border integrity of the canal is preserved was determined to be the end of the canal. The sagittal, axial, and coronal planes also examined the cortex's integrity. All the parameters listed below were measured:

Mediolateral Diameters Superior (MLDS): The mediolateral diameters of the bony nasolacrimal canal in the section where the canal begins in the axial plane (Fig. 1).

Antero-posterior Diameter Superior (APDS): The anteroposterior diameter of the bony nasolacrimal canal in the section where the canal begins in the axial plane (Fig. 1).

Distance Between Canal and Outer Cortex Superior (DCCS): The distances between the anterior border of the nasolacrimal canal and the outer cortical surface of the maxilla in the section where the canal begins in the axial plane (Fig. 1).

**Figure 2:**
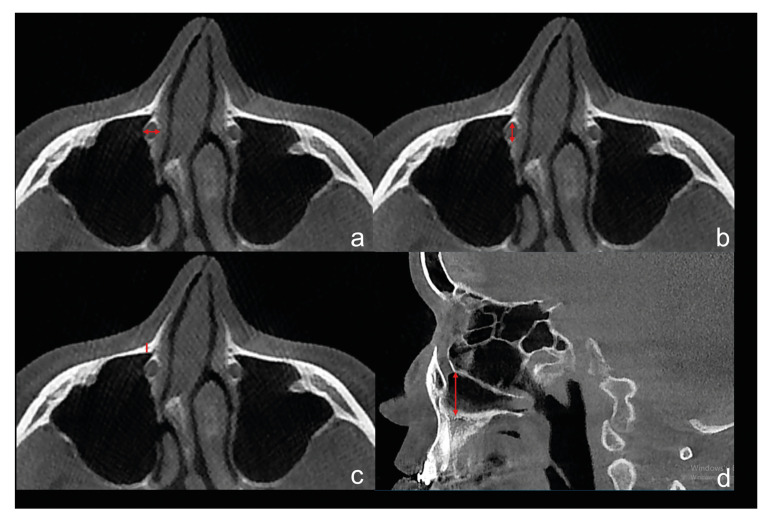
Measurement of the end of the bony nasolacrimal canal (inferior level) on preoperative CBCT images a: The mediolateral diameters of a bony nasolacrimal canal in the section where the canal ends in the axial plane, b: The anteroposterior diameter of the bony nasolacrimal canal, c: The distances between the anterior border of the nasolacrimal canal and the outer cortical surface of the maxilla, d: The distance between most inferior cortical border of nasolacrimal canal and the nasal floor.

Mediolateral Diameter (MLD): The mediolateral diameters of a bony nasolacrimal canal in the section where the canal ends in the axial plane (Fig. 2).

Antero-posterior Diameter Inferior (APDI): The anteroposterior diameter of the bony nasolacrimal canal in the section where the canal ends in the axial plane (Fig. 2).

Distance Between Canal and Outer Cortex Inferior (DCCI): The distances between the anterior border of the nasolacrimal canal and the outer cortical surface of the maxilla in the section where the canal ends in the sagittal plane (Fig. 2).

Distance Between Canal and Nasal Floor (DCN): The distance between the most inferior cortical border of the nasolacrimal canal and the nasal floor. The distance measured in the sagittal plane between the line tangent to the floor of the nasal fossa in the coronal plane and the anterior border of the canal at the canal's end (Fig. 2).

- Evaluation of the postoperative radiological images

**Figure 3:**
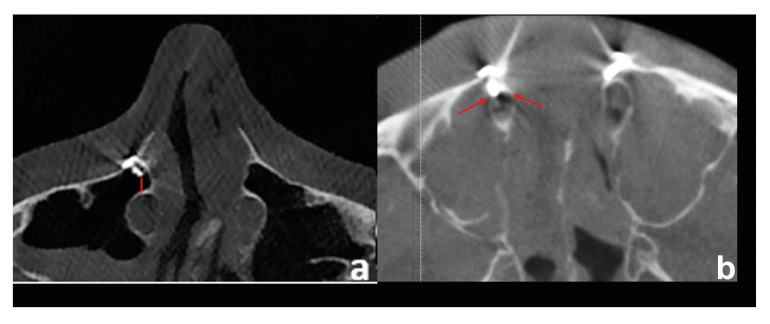
Postoperative CBCT images (a: The distance between the canal and the screws, b: The perforation of the bony nasolacrimal canal).

The same observer repeated all measures three times. To ensure standardization in all images, initially, the FH plane was aligned parallel to the floor in the sagittal plane, and the line passing through the bilateral frontozygomatic suture was also aligned parallel to the floor in the coronal plane. The level of the fixation screws placed in the aperture region was evaluated as "below" in patients located below the lower end point of the bony nasolacrimal canal and "above" in patients located above it. In patients with screw level "above," the distance between the top fixation screw placed in the aperture region and the bony nasolacrimal canal was measured in the axial section. Standard 5-mm mini-screws were used for fixation in all patients. The distance between the canal and the screws was named "DCS" (Fig. 3). The cortical integrity of the nasolacrimal duct was also evaluated in the same section, and the presence or absence of perforation was recorded (Fig. 3).

- Statistical analyses

The normal distribution of the data was evaluated by the histogram, q-q graphs, and Shapiro-Wilk test. The Levene test was used to assess variance homogeneity. The Mann-Whitney U test was used for quantitative variables between the two groups. A Pearson chi-squared analysis was used to compare categorical data. In the present study, evaluated according to Cohen and Evans classifications, intra- and inter-observer correlation and agreement were strong. All data were analyzed by Turcosa Cloud (Turcosa Ltd., Turkey) statistical software. Differences were considered significant at *p* < 0.05.

## Results

A total of 290 nasolacrimal canals were evaluated in 145 patients: 87 females, and 58 males. The mean age was 23.47± 6.67 [18-34]. 30 of the 145 patients exhibited Angle skeletal class 2 deformities, whereas the remaining 115 had Angle skeletal class 3 deformities. There is no statistically significant relationship between gender, age, deformity type, and nasolacrimal canal perforation (Table 1) (*p*>0.05). The anatomical measurements of the nasolacrimal canal (n = 290) are shown in Table 2. There was no statistically significant difference in anatomical measurements between the left and right nasolacrimal canals (*p*>0,05).

**Table 1:**
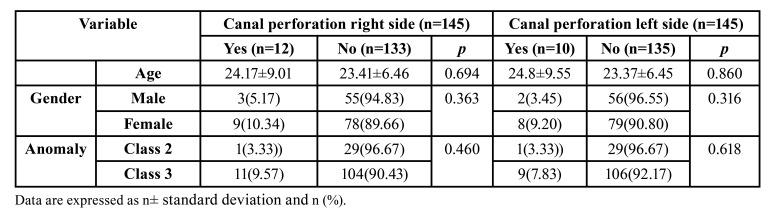
The relationship between demographic variables and nasolacrimal canal perforation.

**Table 2:**
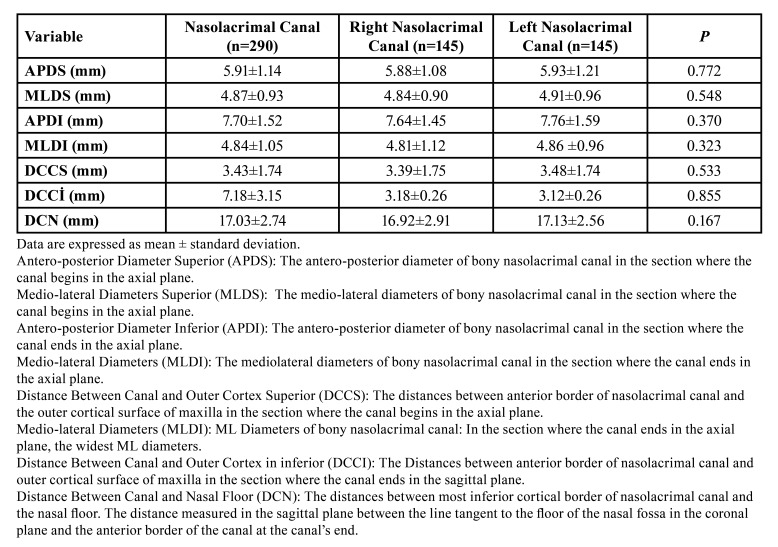
Anatomical measurements of nasolacrimal canal.

The nasolacrimal canal perforation was observed in 22 of the 290 nasolacrimal canals. Hemolacria has been observed in only two patients and was treated conservatively after an ophthalmology consultation. There was no evidence of permanent NLDO in any of the patients. On the 182 sides, the most superior fixation screws were above the inferior opening of the nasolacrimal canal. Nasolacrimal duct perforation was observed in 22 of them. On the 108 sides, the mini screws are positioned below the inferior opening of the nasolacrimal duct. There was a statistically significant relationship between screw level and nasolacrimal canal perforation (*p*<0.001). There was no statistically significant difference in the prevalence of canal perforation between the left and right nasolacrimal canals (Table 3).

The distribution of anatomical measurements between the nasolacrimal canal perforation groups is shown in Table 4. The distance between the most anterior border of the nasolacrimal canal and the outer cortical of the maxilla was statistically significantly less in the group with canal perforation (*p*<0,001). Other anatomical measurements did not differ statistically between the two groups (*p*>0.05). The distance of the screws placed in the aperture region from the most anterior point of the nasolacrimal canal was 0.21 mm ±0.85 mm in the perforated group and 3.60 mm ±1.85 mm in the non-perforated group. The fixation screw was significantly closer to the nasolacrimal canal in the perforation group (*p*<0,001).

**Table 3:**
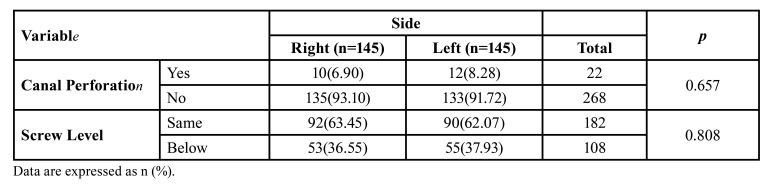
Comparison of nasolacrimal canal perforation and screw level between the left and right sides.

**Table 4:**
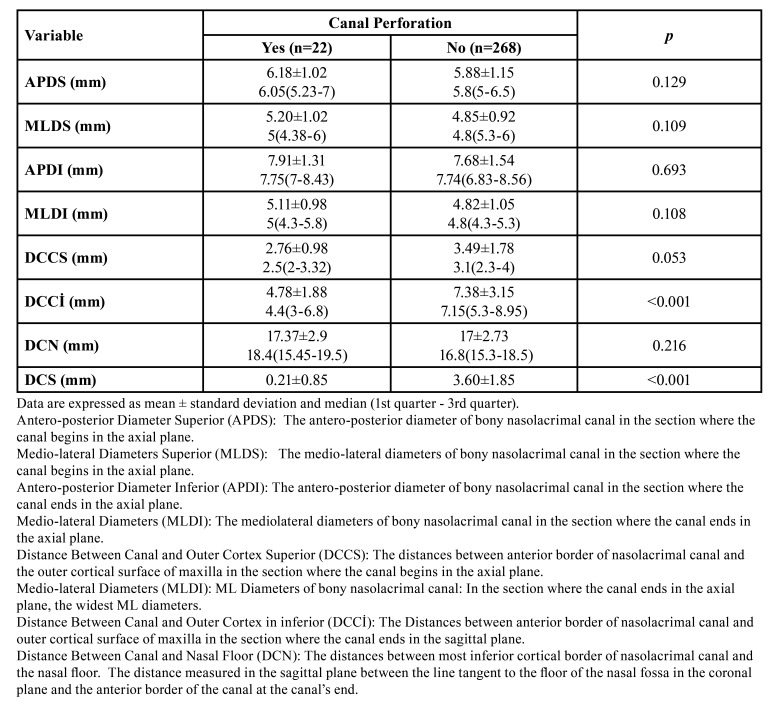
The distribution of anatomical measurement between the nasolacrimal canal perforation groups.

## Discussion

Nasolacrimal duct injury is a rare complication that can occur during the Le Fort I osteotomy. The most common causes of iatrogenic nasolacrimal canal damage during orthognathic surgery include high Le Fort I osteotomies, inferior turbinectomy, instrumentation, and the placement of osteosynthesis screws. It usually occurs during the osteotomy or placement of the osteosynthesis screws (7). This is the first study in which the relationship between fixation screws and drills placed during osteosynthesis and the nasolacrimal canal was evaluated. It was aimed at determining the risk factors arising from the anatomical features of the nasolacrimal duct during Le Fort I surgery. The hypothesis was supported, and the study found that nasolacrimal canal damage may occur during the maxillary fixation procedure of the Le Fort I operation.

A total of 15 cases of nasolacrimal duct obstruction have been reported in the literature after orthognathic surgery (9-13). Keller and Sather reported a case of bilateral epiphora lasting 12 months after quadrangular Le Fort I in a case series of 54 patients (9). Shoshani *et al*. described a patient who had epiphora and dacryocystitis following a Le Fort I osteotomy (10). In the Lanigan *et al*. study, partial turbinectomy and septoplasty were performed to remove edema and the septal deviation causing NLDO (11). Ten of the 15 reported cases were treated with NLDO dacryocystorhinostomy (7). In previous studies, NLDO has often been associated with high Le Fort I osteotomies and inferior turbinectomy. You *et al*. investigated the relationship between high Le Fort I osteotomy and nasolacrimal canal injury in 100 dry skull specimens (14). They reported that the risk of nasolacrimal canal damage was low even if a high Le Fort I osteotomy was performed. In an anatomical study on 15 cadavers, Demas and Sotereanos reported that the inferior opening of the nasolacrimal canal was 11-17 mm above the nasal floor and 11-14 mm posterior to the piriform bone (15). In addition, they performed superior repositioning of the maxilla with a Le Fort I osteotomy in a case series of 34 patients. They concluded that no complication related to the nasolacrimal canal injury developed after 1-3 years of follow-up. In cadaveric research, Little *et al*. revealed that permanent epiphora may occur as a result of plastic deformation in the bony components of the nasolacrimal canal (16). They reported that temporary epiphora occurred because of occlusion of the Hasner valve due to postoperative edema. Özcan *et al*. investigated the prevalence of nasolacrimal canal obstruction and epiphora following maxillary orthognathic surgery (7). In the examination of the postoperative records of 83 patients, symptoms of nasolacrimal duct obstruction were reported in 3 patients, which lasted an average of 32.7 days. NLDO caused by mucosal oedema was observed in two patients, and NLDO in one patient was related to the proximity of the fixation screws to the canal. None of the patients required further surgical treatment of the nasolacrimal duct obstruction. They stated that after Le Fort I surgery, the obstruction of the nasolacrimal duct occurred secondary to nasal packing, an injury causing retrograde inflammation, or mucosal edema and obstruction around the distal orifice. Ozcan *et al*. reported that the meatal opening is approximately 16 mm above the nasal floor, 3 mm in diameter, and usually 30-35 mm behind the lateral margin and anterior nostrils (7). In addition, they performed the standard Le Fort I incision, 8-10 mm above the meatus.

In the present study, a total of 290 bony nasolacrimal canals were evaluated in 145 patients. On the 182 sides, the superior fixation screws were above the level of the inferior opening of the bony nasolacrimal canal. There was a statistically significant relationship between screw level and nasolacrimal canal perforation. There was no statistically significant difference in the prevalence of canal perforation between the left and right nasolacrimal canals. The nasolacrimal canal perforation was observed in 22 of 290 (7,66%) nasolacrimal canals. The distance between the most anterior border of the nasolacrimal canal and the outer cortex of the maxilla was statistically significantly less in the canal perforation group. In addition, the fixation screws were significantly closer to the nasolacrimal canal in the canal perforation group. Although the screws in some cases were not in the nasolacrimal canal, the canal integrity was compromised during the drilling procedure. Despite the loss of bone nasolacrimal canal integrity, NLDO was not observed in all patients due to the nasolacrimal canal's large anterior-posterior diameter. In this study, hemolacria was observed in only two patients, and it was treated with conservative therapy after consultation with ophthalmology. Other anatomical measurements were not statistically different between the two groups. Consistent with the previous study, the most inferior point of the bony nasolacrimal canal measured 17.03±2.74 mm above the nasal floor and 7.18±3.15 mm inside the outer cortex in preoperative CBCT images in this study (7,15).

Various clinical examination methods, as well as imaging procedures such as magnetic resonance imaging, CT, dacryocystography, spiral CT techniques using topical contrast media, and dacrycintigraphy, are used to detect the level of obstruction in nasolacrimal duct obstruction (17-19). In the literature, local massage and the prescription of nasal decongestants, topical antibiotics, and steroid solutions are recommended for the treatment of epiphora signs (10,20). The majority of the previously reported permanent NLDO cases require revision surgery (21). Correct diagnosis, timely initiation, and follow-up of conservative treatment may reduce the need for invasive surgical procedures. In the present study, no patient required surgical intervention for NLDO treatment. The study has some limitations. The first limitation of this study is that the soft tissues of the nasolacrimal duct were not evaluated. The second limitation of the study is the small sample size.

In conclusion, nasolacrimal canal injury may occur during the drilling procedure and insertion of the fixation screws in the aperture region during Le Fort I surgery. Screws positioned most superiorly in the aperture region risk nasolacrimal canal injury. In patients where the nasolacrimal duct is close to the outer cortex, the surgeon should be careful when placing the superior screws in the aperture region to avoid damage to the nasolacrimal duct. NLDO is usually caused by injury-causing retrograde inflammation, mucosal edema, and congestion around the distal orifice. The majority of mucosal edema induced NLDOs are temporary and typically resolve with conservative treatment. The structure of the bone nasolacrimal canal can be determined by preoperative CBCT imaging. Thus, in Le Fort I surgery, osteotomies and osteosynthesis procedures can be customized to the patient to avoid nasolacrimal canal injury. Further studies with large sample sizes are needed to determine risk factors and the incidence of nasolacrimal duct injury.
